# Admixture and natural selection shaped genomes of an Austronesian-speaking population in the Solomon Islands

**DOI:** 10.1038/s41598-020-62866-3

**Published:** 2020-04-23

**Authors:** Mariko Isshiki, Izumi Naka, Yusuke Watanabe, Nao Nishida, Ryosuke Kimura, Takuro Furusawa, Kazumi Natsuhara, Taro Yamauchi, Minato Nakazawa, Takafumi Ishida, Ricky Eddie, Ryutaro Ohtsuka, Jun Ohashi

**Affiliations:** 1 Department of Biological Sciences, Graduate School of Science, The University of Tokyo, Tokyo, 113-0033 Japan; 2 Genome Medical Science Project, Research Center for Hepatitis and Immunology, National Center for Global Health and Medicine, Chiba, 272-8516 Japan; 3 Department of Human Biology and Anatomy, Graduate School of Medicine, University of the Ryukyus, Nishihara, 903-0125 Japan; 4 Graduate School of Asian and African Area Studies, Kyoto University, Kyoto, 606-8501 Japan; 5 Department of International Health and Nursing, Faculty of Nursing, Toho University, Tokyo, 143-8540 Japan; 6 Faculty of Health Sciences, Hokkaido University, Sapporo, 060-0812 Japan; 7 Graduate School of Health Sciences, Kobe University, Kobe, 654-0142 Japan; 8 National Gizo Hospital, Ministry of Health and Medical Services, P.O. Box 36, Gizo, Solomon Islands; 9 Japan Wildlife Research Center, Tokyo, 130-8606 Japan

**Keywords:** Evolution, Genetics

## Abstract

People in the Solomon Islands today are considered to have derived from Asian- and Papuan-related ancestors. Papuan-related ancestors colonized Near Oceania about 47,000 years ago, and Asian-related ancestors were Austronesian (AN)-speaking population, called Lapita, who migrated from Southeast Asia about 3,500 years ago. These two ancestral populations admixed in Near Oceania before the expansion of Lapita people into Remote Oceania. To understand the impact of the admixture on the adaptation of AN-speaking Melanesians in Near Oceania, we performed the genome-wide single nucleotide polymorphism (SNP) analysis of 21 individuals from Munda, the main town of the New Georgia Islands in the western Solomon Islands. Population samples from Munda were genetically similar to other Solomon Island population samples. The analysis of genetic contribution from the two different ancestries to the Munda genome revealed significantly higher proportions of Asian- and Papuan-related ancestries in the region containing the *annexin A1* (*ANXA1*) gene (Asian component > 82.6%) and in the *human leukocyte antigen* (*HLA*) *class II* region (Papuan component > 85.4%), respectively. These regions were suspected to have undergone natural selection since the time of admixture. Our results suggest that admixture had affected adaptation of AN-speaking Melanesians in the Solomon Islands.

## Introduction

The first immigrants into Oceania colonized Near Oceania, which comprises mainland New Guinea and surrounding islands such as the Bismarck Archipelago and the main Solomon Islands, about 47,000 years ago^1^. They are Papuan-related ancestors, non-Austronesian (NAN)-speaking indigenous Melanesians, living in Near Oceania. Probably because of the large ocean lying to the east of Near Oceania, they did not extend into Remote Oceania, which includes all islands in Polynesia and Micronesia. The first colonization of Remote Oceania occurred in the Late Holocene by Austronesian (AN)-speaking people from Southeast Asia. They are called Lapita people after their culture, Lapita, which is characterized by high navigation skills and potteries ﻿decorated with distinctive motifs. Remains of their characteristic pottery suggest that they originated in Taiwan and arrived in the Bismarck Archipelago about 3,500 years ago^2–4^.

Genetic studies of Near and Remote Oceanian populations demonstrated that most Oceanian people had both Asian- and Papuan-related ancestry components; therefore, it is considered that the Lapita people, Asian-related ancestors, admixed with NAN-speaking indigenous people, Papuan-related ancestors, in Near Oceania, before their expansion into Remote Oceania^5–8^. The dates of admixture in various Oceanian populations from the Solomon Islands, Bougainville and Bismarck Archipelago have been shown to mostly fall between 2300 and 3100 years ago^8^.

The Solomon Islands consist of a chain of six large islands (i.e., Guadalcanal, Choiseul, Santa Isabel, New Georgia, Malaita and Makira) and approximately, 900 small islands. In a large-scale study on mitochondrial DNA (mtDNA) and Y chromosome variations in Near and Remote Oceania, haplogroups of Asian origin and Near Oceanian origin are both represented in different populations of the Solomon Islands^6^. In addition, a recent study using a genome-wide dataset of 823 individuals from 72 populations, including 50 populations from Oceania also found signals of admixture in populations from the Solomon Islands^8^. The results of these two studies indicate that people in the Solomon Islands have Asian- and Papuan-related ancestries. Previously we studied mtDNA variations in AN-speaking Melanesian populations in the New Georgia Island, the western province of the Solomon Islands to examine the genetic affinity between AN-speaking Melanesians in the Solomon Islands and AN-speaking Polynesians^9^. The people of Munda, one of the studied populations living in the main town of the New Georgia Islands, had mtDNA haplogroups of both Near Oceanian and Asian origins as found in other populations from the Solomon Islands, suggesting that Munda people also have Asian- and Papuan-related ancestries.

In this study, we investigated the following genetic characteristics of Munda population using the genome-wide single nucleotide polymorphism (SNP) data: genetic relationship between Munda and other Oceanian populations, a sign of the admixture in Munda genomes, and signals of positive selection based on the excess of either Asian-related or Papuan-related ancestry observed in specific genomic regions of Munda. To assess the positive selection (i.e., the effect of admixture on the adaptation of Munda population), we performed a genome-wide analysis of local ancestry of Munda individuals using the Efficient Local Ancestry Inference (ELAI) algorithm^10^, which was recently applied for the detection of potential selection signals in the genomes of Malagasy, an AN-speaking population in Madagascar who have AN-related and Bantu-related genetic ancestries^11^. Our results revealed that: (1) the Munda people were genetically related to other populations from the Solomon Islands, (2) the Munda genomes consisted of both Asian- and Papuan-related ancestry components, and (3) the significant amounts of Asian- and Papuan-related ancestries were observed in the region containing the *annexin A1* (*ANXA1*) gene and in the *HLA class II* region, respectively. These genomic regions are considered to have experienced natural selections since the time of admixture. Although the possibility that these regions were shaped by only genetic drift still remains, we found that positive selection acted over the *HLA class II* region in modern Papuans. This observation indirectly supports the scenario that the *HLA class II* region of the Munda genomes has been subjected to selection. Considering that HLA class II molecules and annexin A1 protein both play important roles in immunity, infectious diseases may have been a strong selective pressure in Munda.

## Results

### Genetic structure in munda indicates their close relationship to other solomon populations

**Figure 1:**
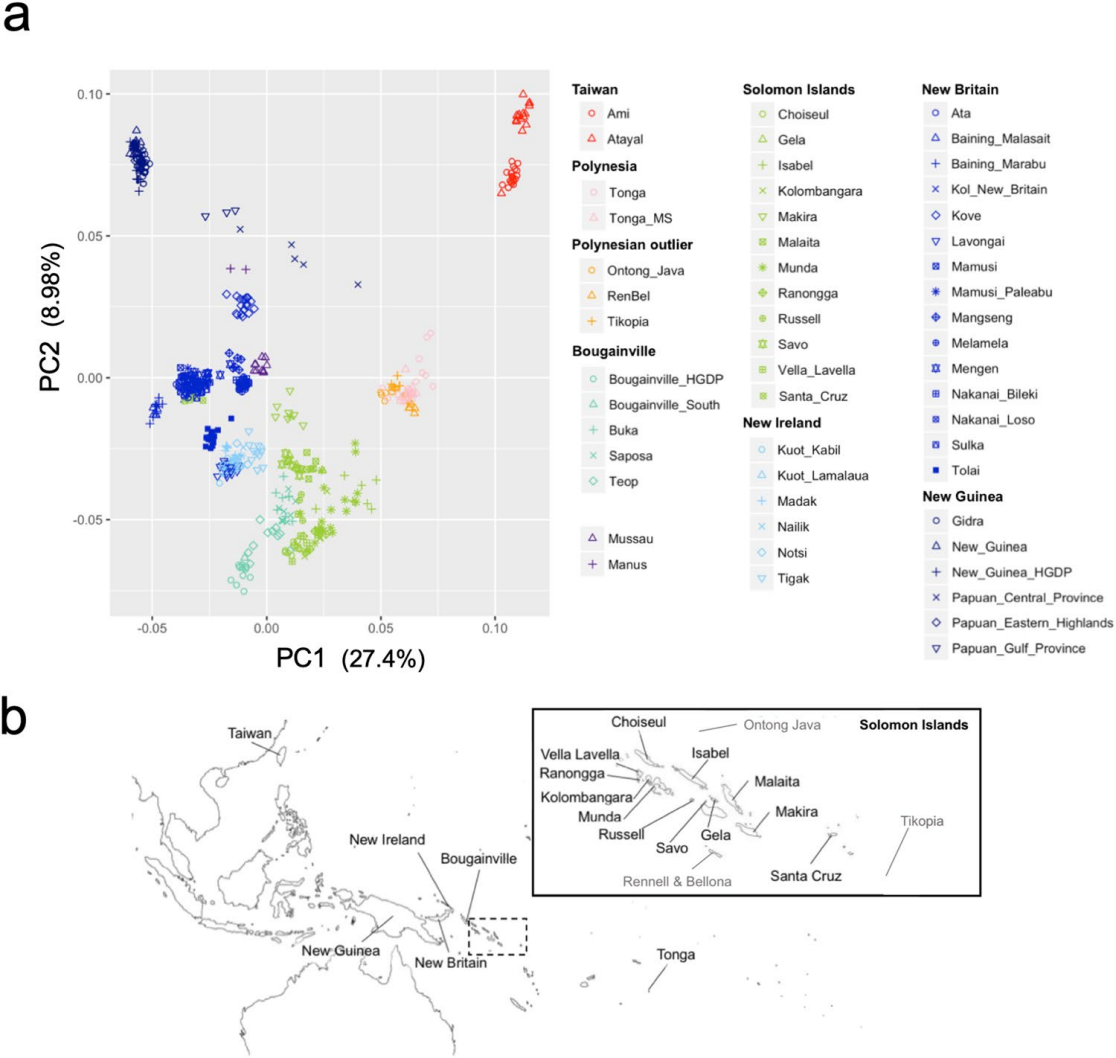
PCA plot for 52 Oceanian populations and two Aboriginal Taiwanese populations (**a**) and geographic locations of Oceanian populations (**b**). (**a**) Each dot represents an individual. The yellow green color labels indicate populations in the Solomon Islands. Munda subjects gather in a cluster with other subjects in the Solomon Islands except for Santa Cruz. The eigenvalues and percentages of variance were 27.4 and 5.08% for PC1 and 8.98 and 1.66% for PC2. (**b**) Populations written in gray in the map of the Solomon Islands were Polynesian outliers.

**Figure 2:**
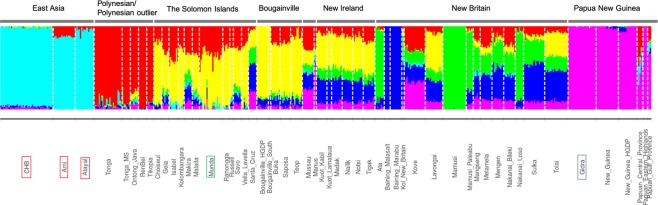
Results of the ADMIXTURE analysis performed on the entire dataset (K = 6). Each individual was divided into six ancestry components. The gray bars above the plot indicate geographic regions and the populations used as proxies for Papuan and Asian ancestries and Munda were marked by squares in blue, red and green, respectively.

A principal component analysis (PCA) plot of 51 Oceanian and two Taiwanese populations is shown in Fig. 1. Geographically adjacent populations were located close to each other (Fig. 1). Populations from the Solomon Islands, except for Santa Cruz, clustered, while Santa Cruz was located in the cluster of New Britain Island (Fig. 1a). Figure 2 illustrates individual ancestry proportion inferred by ADMIXTURE analysis for K = 6, which provided the lowest cross-validation error for Ks ranging from 2 to 8 (Fig. S1). The yellow component, which is present at high frequency in Bougainville and the Solomon Islands, and the red component, which is observed at high frequency in Tongans and Polynesian outliers, accounted for almost all of the inferred ancestries in populations from the Solomon Islands except for Santa Cruz and Makira. These results may indicate close relationship between Munda and other Solomon populations.

### Munda experienced admixture between Asian- and Papuan-related populations ~2.3 kya

Since Asian-related ancestors for the Solomon Islands populations are considered to have originated in Asia, probably Taiwan, we calculated f3 statistics for populations in the Solomon Islands using Han Chinese (CHB) or Aboriginal Taiwanese as a proxy for Asian-related ancestors and Gidra for Papuan-related ancestors to test if Munda population descends from a mixture of the two ancestral populations. Concordant with the previous studies, majority of the population of Solomon Islands, including Munda, showed significantly negative f3-statistics, indicating that they are a result of admixture between Asian- and Papuan-related ancestors (Table 1). The date of admixture was estimated as ~2,300 years ago (77.6 ± 2.2 generations) based on the decline of linkage disequilibrium (Fig. S2) by ROLLOFF software, assuming CHB and Gidra as Asian- and Papuan-related ancestors.

**Table 1:** Results of 3-Population test for the Solomon Islands populations.

Population C	Population A = CHB, Population B = Gidra				Population A = Taiwan, Population B = Gidra			
	f3	std. err	Z	SNPs	f3	std. err	Z	SNPs
Munda	−0.00279	0.000933	**−3.00**	48584	−0.00869	0.000943	**−9.22**	48407
Kolombangara	−0.00442	0.00118	**−3.75**	48269	−0.00932	0.00122	**−7.65**	48149
Choiseul	−0.0167	0.000951	**−17.6**	48286	−0.0206	0.000961	**−21.4**	48156
Ranongga	0.00171	0.00126	1.35	48295	−0.00387	0.00126	**−3.08**	48153
Malaita	−0.00781	0.00107	**−7.30**	48281	−0.0131	0.00105	**−12.4**	48159
Russell	−0.00820	0.00196	**−4.18**	48195	−0.00892	0.00186	**−4.79**	48067
Gela	−0.0121	0.00104	**−11.7**	48285	−0.0169	0.00101	**−16.8**	48153
Savo	−0.00800	0.00108	**−7.43**	48310	−0.0135	0.00108	**−12.5**	48178
Isabel	−0.0118	0.000971	**−12.2**	48324	−0.0157	0.000984	**−15.9**	48179
Vella_Lavella	−0.00049	0.00125	−0.389	48237	−0.00782	0.00119	**−6.58**	48123
Santa_Cruz	−0.0175	0.000923	**−19.0**	48289	−0.0210	0.000925	**−22.76**	48156
Makira	−0.00259	0.00122	−2.13	48415	−0.00756	0.00118	**−6.41**	48257

For estimating f3 statistics, population C (the Solomon Islands populations) was supposed to have descended from a mixture of populations A (Asian-related ancestral population) and B (Papuan-related ancestral population). Z scores less than −2.33, the percentage of values to the left of which is ~1%, were regarded as significantly deviated from 0 (written in bold).

### Genomic components from Asian- and Papuan-related ancestries were almost equal in the munda genomes

**Figure 3:**
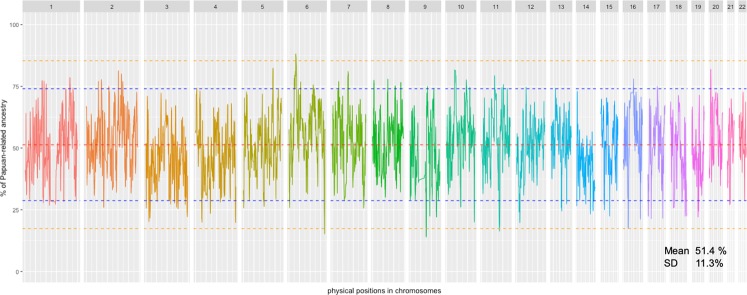
Average Papuan-related ancestry estimated using ELAI across Munda genomes for each genomic position of each autosomal chromosome. Each color represents different chromosome. In this analysis, Gidra and CHB populations were used as proxies for Papuan-related and Asian-related ancestries in Munda subjects. Red dashed line represents the genome-wide mean. Blue and orange dashed lines represent ±2 SD and ±3 SD from the mean, respectively. The genome regions above the upper orange line and below the lower orange line were regarded as the high Papuan- and Asian- related ancestries regions, respectively.

The contribution of Asian- and Papuan-related ancestry across Munda genomes (Papuan versus Asian ancestry) was measured by the ELAI algorithm using CHB and Gidra as proxies for Asian- and Papuan-related ancestors. In this study, instead of Aboriginal Taiwanese, CHB were used as Asian-related ancestors, since the number of SNPs in the dataset including CHB was approximately three times larger than the one including Aboriginal Taiwanese. Figure 3 shows the average proportion of Papuan-related ancestry across each position in Munda genomes estimated by ELAI program setting the admixture generations as 77. The genome-wide average proportions of Asian- and Papuan-related ancestries were 48.6 ± 11.3% and 51.4 ± 11.3%, respectively. We also performed the F4 Ratio Test, originally called the f4 Ancestry estimation in Moorjani *et al*. (2011), assuming a phylogeny shown in Fig. S3. The proportions of Asian-related and Papuan-related ancestries were estimated as 43.1 ± 1.3% and 56.9 ± 1.3%, respectively. These results indicate that genomic components from Asian- and Papuan-related ancestries were almost equal in the Munda genomes.

### Excessive proportions of Asian- and Papuan-related ancestries were observed in the genomic regions involved in immunity

The proportion of genetic ancestries is expected to be approximately normally distributed across the genome, when only random genetic drift operates. If natural selection acts, the values of the genetic region targeted by selection would be outliers in the distribution. Thus, if an excess of local ancestry related to either of parental group is observed in the specific genome region, the region is regarded as a potential target of natural selection. As seen in Fig. 3, the genomic positions with the highest Asian-related ancestry and the highest Papuan-related ancestry were found at chr 9: 75695677–75705507 (86.0%) and chr 6: 32430975 (88.1%), respectively. Grubbs’ test indicated that both the positions were outliers of the observed distribution of the mean proportion across genome (*P* < 2.2 × 10^−16^). The former position was located in the high Asian-related ancestry region spanning ~1.4 Mb on chromosome 9 (chr 9: 74835176–76273853) and the latter was in the high Papuan-related ancestry region spanning ~1.8 Mb on chromosome 6 (chr 6: 32233886–33976742). The proportions of Asian- and Papuan-related ancestries in these regions fell outside 3 standard deviations (SDs). The high Asian-related ancestry region (>82.6%) contained guanine deaminase (GDA), zinc finger AN1-type containing 5 (*ZFAND5*), transmembrane channel like 1 (*TMC1*), aldehyde dehydrogenase 1 family member A1 (*ALDH1A1*) and annexin A1 (*ANXA1*), and the high Papuan-related ancestry region (>85.4%) contained human leukocyte antigen (*HLA*) class II genes, such as *HLA-DRB1, HLA-DQA1, HLA-DPA1* and *HLA-DRA*. Although the genes directly targeted by natural selection are hard to be inferred, annexin A1 protein and HLA class II molecules play important roles in immunity.

To investigate the effect of the assumption about the generation on the proportion of the Papuan-related component at each position, ELAI analyses were conducted with setting generations since the time of admixture as 50, 100 and 150. The highest position (chr 6:32430975) was consistent (Fig. S4), while the lowest position was slightly changed when the generations assumed were changed but always located within the low Papuan-related ancestry region on chromosome 6 (Fig. S5). If a genome region derived from either of the ancestries had undergone positive selection since the time of admixture, the sign could be detected broadly because the admixture occurred just a few thousand years ago. The regions detected on chromosomes 6 and 9 spanned more than 1.0 Mb and consistently represented proportions outside 3 SD regardless of assumed date of admixture, although there were some other regions showing the proportions outside 3 SD (Fig. S5).

### Distribution of local ancestry was estimated by a coalescent simulation under neutrality

To examine if the deviations from the mean proportion observed on chromosomes 6 and 9 were caused by only genetic drift, we further conducted ELAI analysis similarly for genotype data generated by coalescent-based simulations under the assumption of selective neutrality. The distribution of local ancestry obtained from the simulation data was quite similar to that of real data (Fig. S6a,b). The mean (51.7%) and SD (11.6%) were compatible to those of real data (Mean = 51.4% and SD = 11.3%). Our coalescent simulation generated genomic positions with the proportion of either of ancestry 3 SD away from the mean, suggesting that genetic drift can produce the proportion of ancestry outside 3 SD. However, the lengths of the regions that continuously have proportions of Papuan-related ancestry larger than +3 SD or smaller than −3SD in the simulation were not as long as those observed in our real data (1.8 Mb on chromosome 6 and 1.4 Mb on chromosome 9); the length in the simulation was 400 kb at most. The rate of recombination in the *HLA* region is known to be lower than the average rate of the human genome^12^. The lower recombination rate may have resulted in more extended linkage disequilibrium (LD). Therefore, the simulation, assuming the recombination rate of the corresponding *HLA* region (i.e., a recombination rate of 8.5 × 10^−9^/base/generations), was also conducted. The average length of the regions that continuously have proportions of Papuan-related ancestry larger than +3 SD was 490 kb, and the maximum length was 1.1 Mb in the simulation (Fig. S6c). The maximum length, 1.1 Mb, was shorter than the observed one, 1.8 Mb, for the *HLA* class II region. Therefore, the excessive proportion of Papuan-related ancestries observed in the *HLA* class II region seems to have been caused by natural selection.

### Strong signatures of recent positive selection were observed in the *HLA class II* region in the modern papuans

**Figure 4:**
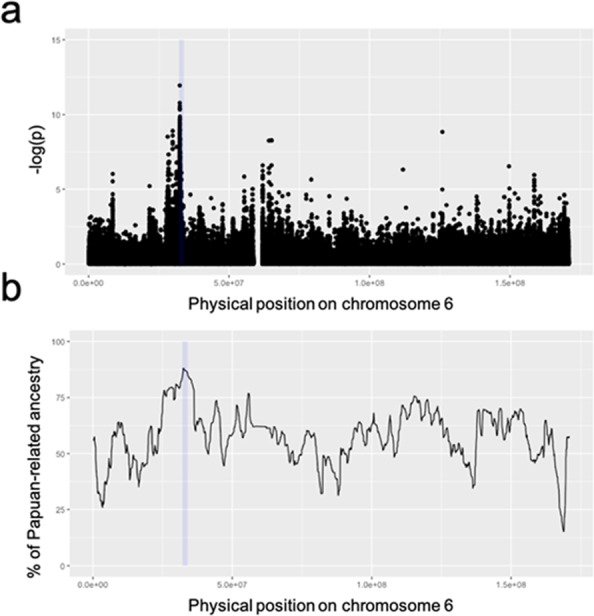
Manhattan plot of iHS P-values across chromosome 6 in modern Papuans (**a**) and average Papuan-related ancestry estimated using ELAI across chromosome 6 in Munda genomes (**b**). (**a**) The y-axis denotes the negative logarithm of P-values. (**a**,**b**) The high Papuan-related ancestry region was shaded in blue.

If the increased Papuan-related ancestry results from recent positive selection against the genomic components derived from Papuans, a signal of positive selection may be found on the same region in the modern Papuans, who shared similar environment with populations in the Solomon Islands. To examine this assumption, the integrated Haplotype Score (iHS)13, developed for detecting recent positive selection based on the degree of extended haplotype homozygosity (EHH), was calculated across chromosome 6 in modern Papuans (n = 14). The most significant signal (i.e., the lowest *P*-value) was observed at the position of chr 6:32487913 (rs200439840; iHS = 7.11, *P* = 1.13 × 10^−12^), and SNPs with low *P*-values were accumulated in the *HLA class II* region (Fig. 4a). This region coincided with high Papuan-related ancestry region in Munda genomes (Fig. 4b).

## Discussion

A PCA plot for Oceanian populations (Fig. 1a) reflected geographic locations of each population sample consistent with the previous study^8^ and admixture analysis showed the Solomon Islands population samples including Munda shared similar ancestry components (Fig. 2). The Solomon Islands populations were considered to have experienced admixture based on lines of genetic evidence^5–8^. Recently Pugach *et al*.^8^ estimated f3 statistics and found signals of admixture for the Solomon Islands populations. Concordant with their results, we found significant signals of admixture for Munda (Table 1), when we assumed Aboriginal Taiwanese or CHB as Asian-related ancestry populations and Gidra as a Papuan-related ancestry population. The proportions of Papuan-related ancestry estimated with different methods (ELAI and F4 Ratio Test) fell between 51–57% in Munda. Although the admixture date estimates by ROLLOFF tend to be biased for old mixture dates, small mixture proportions or small sample sizes^13^, the admixture date (~2,300 years ago) of Munda estimated in this study fell within the range of admixture date (∼2,300–3,100 years ago) of various Oceanian populations estimated by Pugach *et al*.^8^. These results suggest that Munda share population history with other AN-speaking populations in the Solomon Islands.

The ELAI analysis detected the regions with significantly high amounts of Asian- and Papuan-related ancestries in the Munda genomes. These regions were considered to have been shaped by natural selection based on the principles that have been used to detect “adaptive introgression” of genes in admixed populations^10,11,14^. Although the coalescent simulation revealed that genetic drift could cause the excess of either of local ancestry to the observed extent, the maximum length of the regions that continuously have proportions of Papuan-related ancestry larger than +3 SD or smaller than −3 SD in the simulation was shorter than the observed ones (1.8 Mb in *HLA* class II region and 1.4 Mb in the *ANXA1* region). The extended LD is one of signatures of recent positive selection^15^. Thus, the co-existence of long-range LD and significant excess of local ancestry in real data could be interpret as a sign of positive selection. Besides, a signal of recent positive selection was independently detected in the *HLA class II* region in modern Papuans (Fig. 4). This corroborates that the region was essential to the adaptation to Oceanian environment and it is reasonable to infer that positive selection also acted on this region in Munda since the time of admixture. Therefore, the two detected regions, at least the high Papuan-related ancestry region, are likely to have been shaped by positive selection in Munda. A further analysis by increasing Munda subjects would elucidate if these regions have been shaped by genetic drift or positive selection.

The high Papuan-related ancestry region contained the *HLA class II* genes coding HLA molecules, which play central roles in the adaptive immune system by presenting peptides derived from extracellular proteins to T-lymphocytes. The *HLA class II* genes have more than 5,000 alleles, of which alleles of the *HLA-DRB1* account for nearly half (2,268 alleles), according to the IPD-IMGT/HLA Database^16^. Additionally, the *HLA* polymorphisms were considered to be formed by pathogen-driven balancing selection^17^. For example, a previous study on the *HLA-DRB1* diversity for a Mongolian population who moved from the north to the south of China indicated that the difference of pathogens in the environment could alter the frequency of the *HLA-DRB* haplotypes^18^. New Guinea and the Solomon Islands belong to tropical regions and a number of tropical infectious diseases such as malaria have been prevalent until today^19^. The pathogen-rich environment may have influenced the *HLA* polymorphisms of populations in New Guinea and the Solomon Islands over tens of thousands of years and the *HLA* haplotype variations in Papuan-related ancestors would have been advantageous for the pathogen-rich environment better than those in Asian-related ancestors. Corresponding with the above scenario, we hypothesized that the adaptive *HLA* haplotype variations derived from Papuan-related ancestors have been adaptively conserved even after admixture, which resulted in high proportion of Papuan-related ancestry in the *HLA class II* region in Munda. Our hypothesis is consistent with a previous study^20^ that reported similar allele frequencies of *HLA-DRB1* among NAN-speaking Melanesians, AN-speaking Melanesians and Polynesians. In addition, several studies that investigated the distribution of local ancestries also reported signatures of natural selection on the *HLA* regions in admixed populations; an excess of African ancestry in the *HLA* regions was found in Mexicans and Latino populations recurrently^10,14,21^. The *ANXA1* gene, which is present in the high Asian-related ancestry region, encodes a membrane-localized protein that binds phospholipids and is also known to have functions involved in innate and adaptive immune systems^22,23^. One of the possible driving forces of the selection that acted over the two genomic regions of Munda may be infectious disease.

## Materials and Methods

### Subjects

Blood was sampled in Munda, the main town of New Georgia Island, after obtaining informed written consent from each participant. All methods were performed in accordance with the relevant guidelines and regulations. This study was approved by the Health Research Ethics Committee, Ministry of Health, Solomon Islands, the Ministry of Education and Training, Solomon Islands, and the Research Ethics Committees of the Faculty of Science, and the Faculty of Medicine, The University of Tokyo.

### Genome-wide SNP data

Genomic DNA was extracted from peripheral blood using a QIAamp Blood Kit (Qiagen, Hilden, Germany). SNP genotyping was performed for 21 individuals from Munda with the Affymetrix GeneChip® Human Mapping 250 K Nsp SNP array according to the protocol described in a previous study^24^. Next, we obtained a genome-wide dataset comprising 21 individuals from Munda population and 231,049 autosomal SNPs. Although all individuals were sampled as unrelated, we checked Identical-By-Descent (IBD) values of each pair of samples. The calculation of IBD values was performed after LD pruning using PLINK software v1.90b5.2 (www.cog-genomics.org/plink/1.9/)^25^. LD pruning was performed with the following settings, which define window size, step and the r2 threshold:–indep-pairwise 50 5 0.5. As a result, the number of SNP markers was reduced to 69,577. Since one pair of individuals showed IBD value higher than 0.2 (IBD value = 0.4728), we excluded one individual in that pair in 3-Population Test^26^, F4 Ratio Test^26^ and Effective Local Ancestry Inference analysis (Guan 2014) but retained the individual in Principal component analysis (PCA), ADMIXTURE^27^ analysis and admixture time estimation using ROLLOFF^13^.

### PCA and ADMIXTURE Analysis

We merged the genome-wide dataset of Munda population obtained in this study with previously reported dataset comprising 24 individuals from Tonga, AN-speaking Polynesians, and 24 individuals from Gidra, NAN-speaking Melanesians in the lowlands of Western Province, Papua, New Guinea (393,971 autosomal SNPs)^24^, and a dataset of 443 individuals from 48 Oceanian populations and two Taiwanese populations extracted from the genome-wide dataset comprising 823 individuals and over 620,000 autosomal SNPs^8,28–30^. After merging the datasets using PLINK software, a genome-wide dataset comprising 512 individuals from 53 populations and 49,523 autosomal SNPs were obtained (dataset 1). The list of the populations used in this study is shown in Supplementary Table 1. A principal component analysis (PCA) was performed on dataset 1 using PLINK software. ADMIXTURE analysis was conducted by ADMIXTURE version 1.3.0 for different values of K (from K = 2 through K = 8) on a dataset in which the HapMap data of 45 unrelated individuals from Han Chinese from Beijing (CHB)^31^ were further added to dataset 1 (dataset 2). Cross-validation procedure implemented in ADMIXTURE package was performed to find the best value of K. The results were drawn using POPHELPER Structure Web App v1.0.10^32^.

### 3-Population test, F4 Ratio Test and admixture date estimation

3-Population Test, F4 Ratio Test and admixture time estimation by ROLLOFF were conducted using the AdmixTools package version 4.1^26^. To test if the admixture occurred in Munda populations, the 3-Population Test^26^ was conducted. The f3(C; A, B) statistics should be negative if a population C has descended from a mixture of populations A and B. Assuming CHB and Gidra as a proxy of Asian-related and Papuan-related ancestors, respectively, f3(C; CHB, Gidra) was calculated; population C was assumed to be one of the Solomon Islands populations. The f3-statistics were also calculated assuming Aboriginal Taiwanese (Atayal and Ami) as a proxy of Asian-related ancestors instead of CHB since Lapita people are suspected to have originated in Taiwan^33^. The numbers of SNPs used for f3 calculation are listed in Table 1. A one-tailed test was performed for f3-statistics using the Z score. The Z score less than −2.33 was regarded as statistically significant (the significance level was set to be 0.01).

To estimate the proportion of admixture, we conducted F4 Ratio Test assuming the population relationships shown in Fig. S3. The proportion of Asian- and Papuan-related ancestries, α and 1-α, respectively, was estimated by computing the ratio of two f4 statistics: α = f4 (CHB, YRI; Munda, Gidra)/f4 (CHB, YRI; Taiwan, Gidra)^26,34^. The dataset 2 and the HapMap data of 60 unrelated individuals from Yoruba in Ibadan, Nigeria (YRI)^31^ were also used for this analysis.

ROLLOFF analysis^13^ implemented in Admixtools package^26^ was performed for a genome-wide dataset comprising 21 individuals of Munda, 24 individuals from Gidra and 45 individuals from CHB and 231,049 autosomal SNPs (dataset 3) to estimate the date of admixture experienced by the ancestors of Munda. In brief, ROLLOFF estimates the date of admixture based on the rate of exponential decline of admixture-induced linkage disequilibrium. Genetic distance (cM) between SNPs was determined assuming that 1 cM was equal to 1 Mb. Next, ROLLOFF analysis was conducted with the settings which define a bin size as 0.1 cM and maximum distance as 20 cM.

### Detecting signals of positive selection after admixture

To detect signals of positive selection that might have operated after admixture, we conducted ELAI analysis across Munda genomes. Using PLINK software, one individual with high IBD value was excluded from dataset 3 and SNPs were filtered by genotyping rate higher than 0.95 in each population. The final dataset (dataset 4) used for ELAI comprised 89 individuals and 146,090 autosomal SNPs. ELAI analysis was performed with the ELAI version 1.00 software with the settings which define the number of EM steps as 20, the upper layer number of clusters as 2, and lower layer number of clusters as 10 according to the manual^10^. The admixture generations were set as 77 based on the dates of admixture for the Solomon Islands populations estimated in this study. In addition, to investigate the effect of assumed generations since the admixture on the local ancestry estimation, the dates of admixture were set as 50, 100 and 150 generations ago, which covered the whole range of the previously estimated date of admixture in Oceanian populations: the dates of admixtures fell between 3,300-1,800 years ago with 95% confidence intervals ranged from ~1,600 to more than 3,600 years ago assuming a generation time of 30 years^8^. Statistical analysis was conducted using R version 3.5.3 and the mean values of local ancestry across the Munda genomes were plotted using R package ggplot2 version 3.1.1^35^. To detect outliers on opposite tails, we conducted Grubbs’ test on the mean values of local ancestry across each position in Munda genome using R package “outliers“ version 0.14^36,37^.

### Coalescent-based simulations

We used coalescent simulations to address if genetic drift alone could produce observed patterns of admixture in the Munda genomes. Coalescent-based simulations were performed using R package “scrm” version 1.7.3.1^38^. To reproduce the population history of Gidra, CHB and Munda, simulations were performed assuming the following population history. First, two subpopulations (Anc1 and Anc2) diverged from one ancestral population 1667 generations ago, which corresponds to 50,000 years ago when generation time is 30 years. Next, subpopulations Anc1’ and Anc2’diverged from Anc1 and Anc2, respectively 77 generations ago (2,310 years ago). Then, these subpopulations were mixed at the same generation, 77 generations ago (Fig. S7). The descendants of Anc1 and Anc2 were regarded as Gidra and CHB, respectively, and the admixed population was regarded as Munda population. Segregating sites within 2Mb-long sequence were sampled 1,500 times (i.e., the total length corresponded to 3 Gb) for 48, 40 and 90 chromosomes from hypothetical Gidra, Munda and CHB populations, respectively, assuming a mutation rate of 1.2 × 10^−8^/base/generations and a recombination rate of 1.3 × 10^−8^/base/generations^39–41^. In addition, a recombination rate of 8.5 × 10^−9^/base/generations, which corresponded to the *HLA* region, was also assumed. The recombination rate in the *HLA* region was obtained from the HapMap database^42^. The size of each population was assumed to be 1,000. The average genome-wide admixture rate estimated by ELAI analysis (0.5137) was used as the admixture rate. Next, the genotype data for 24, 20 and 45 individuals from hypothetical Gidra, Munda and CHB populations were generated from the obtained sequences. Considering SNP ascertainment bias observed in real data, 146,090 SNPs, that exhibited the same distribution of minor allele frequencies as the real data, were randomly extracted from the simulated genotype data. ELAI analysis was then conducted with the same settings as described above.

### Detecting signals of positive selections in modern papuans

To detect signals of natural selection in modern Papuans, integrated haplotype scores (iHS)^43^ across the whole chromosome 6 were calculated for modern Papuans (n = 14) in Papua New Guinea investigated in the Simons Genome Diversity Project^44^. The data were filtered by PLINK software with the following settings which define the minimum of minor allele frequency (maf), threshold of missing calls per variant, threshold of missing calls per sample, excluding variants with multi-character allele, and including only biallelic sites: –maf 0.01–geno 0.05–mind 0.05–snps-only–biallelic-only. Information on ancestral alleles was obtained from 1000 Genomes Project Phase 3^45^. After excluding variants without ancestral allele information, 295,531 SNPs were remained and the genotype data were phased with Beagle 5.0 (beagle.28Sep18.793)^46^. The phased genotype data were used to calculate iHS with R package rehh version 2.0.4^47^.

### Web Resources

Additional studies and information can be found at POPHELPER, http://www.pophelper.com, Outliers, http://CRAN.R-project.org/package=outliers, The IPD and IMGT/HLA database, https://www.ebi.ac.uk/ipd/imgt/hla/, UCSC Genome Browser, https://genome.ucsc.edu/index.html, PANTHER, http://www.pantherdb.org, and HapMap Phase II recombination rate, ftp://ftp.ncbi.nlm.nih.gov/hapmap/recombination/2011-01_phaseII_B37/.

## Supplementary information

Supplementary Information.
