# Supplementary material for: Admixture and natural selection shaped genomes of an Austronesian-speaking population in the Solomon Islands
Source: Sci Rep. 2020 Apr 23;10:6872. doi: 10.1038/s41598-020-62866-3 (PMC7181741; doi:10.1038/s41598-020-62866-3)
Supplement: Supplementary file 1 — Supplementary Information. [file 41598_2020_62866_MOESM1_ESM.pdf]

Supplemental Info (seven figures and one table)

Admixture and natural selection shaped genomes of an Austronesian-speaking population in the Solomon Islands

Mariko Isshiki,[1] Izumi Naka,[1] Yusuke Watanabe,[1] Nao Nishida,[2] Ryosuke Kimura,[3] Takuro Furusawa,[4] Kazumi Natsuhara,[5] Taro Yamauchi,[6] Minato Nakazawa,[7] Takafumi Ishida,[1] Ricky Eddie,[8] Ryutaro Ohtsuka,[9] Jun Ohashi[1, *]

[1]Department of Biological Sciences, Graduate School of Science, The University of Tokyo, Tokyo 113-0033, Japan
[2]Genome Medical Science Project, Research Center for Hepatitis and Immunology, National Center for Global Health and Medicine, Chiba 272-8516, Japan
[3]Department of Human Biology and Anatomy, Graduate School of Medicine, University of the Ryukyus, Nishihara 903-0125, Japan
[4]Graduate School of Asian and African Area Studies, Kyoto University, Kyoto 606-8501, Japan
[5]Department of International Health and Nursing, Faculty of Nursing, Toho University, Tokyo 143-8540, Japan
[6]Faculty of Health Sciences, Hokkaido University, Sapporo 060-0812, Japan
[7]Graduate School of Health Sciences, Kobe University, Kobe 654-0142, Japan
[8]National Gizo Hospital, Ministry of Health and Medical Services, P.O. Box 36 , Gizo , Solomon Islands
[9]Japan Wildlife Research Center, Tokyo 130-8606, Japan

[*]Correspondence: juno-tky@umin.ac.jp

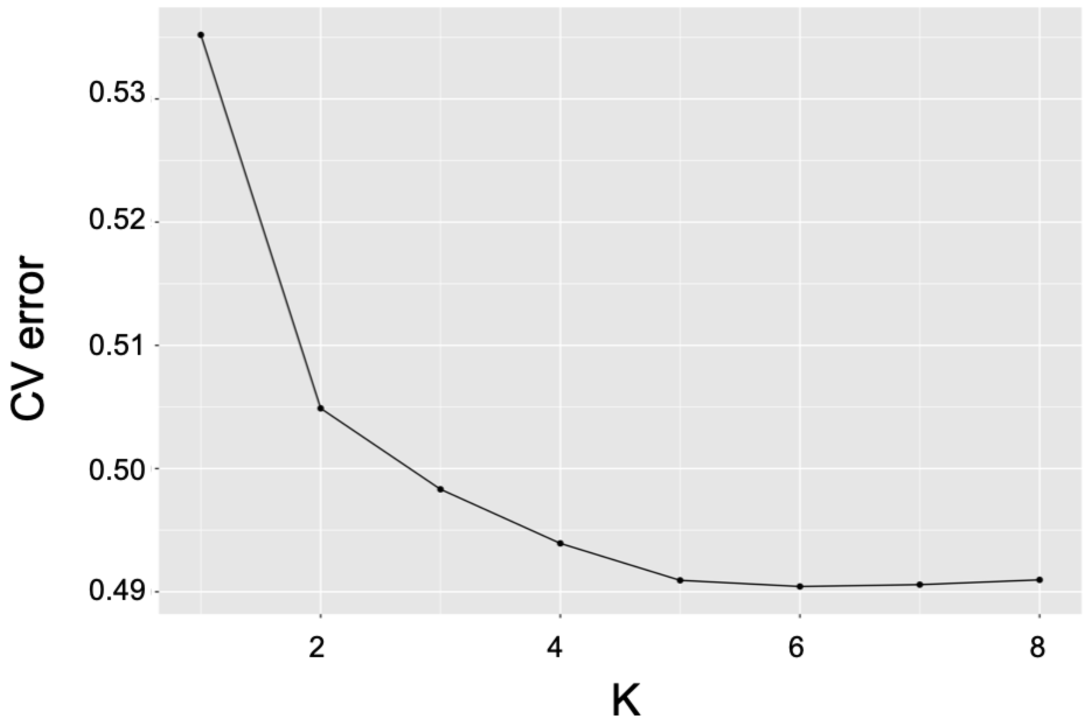

Figure S1. Estimated cross validation error for the ADMIXTURE analysis for the entire dataset (dataset 2).
The result of K =6 is shown in Figure 2.

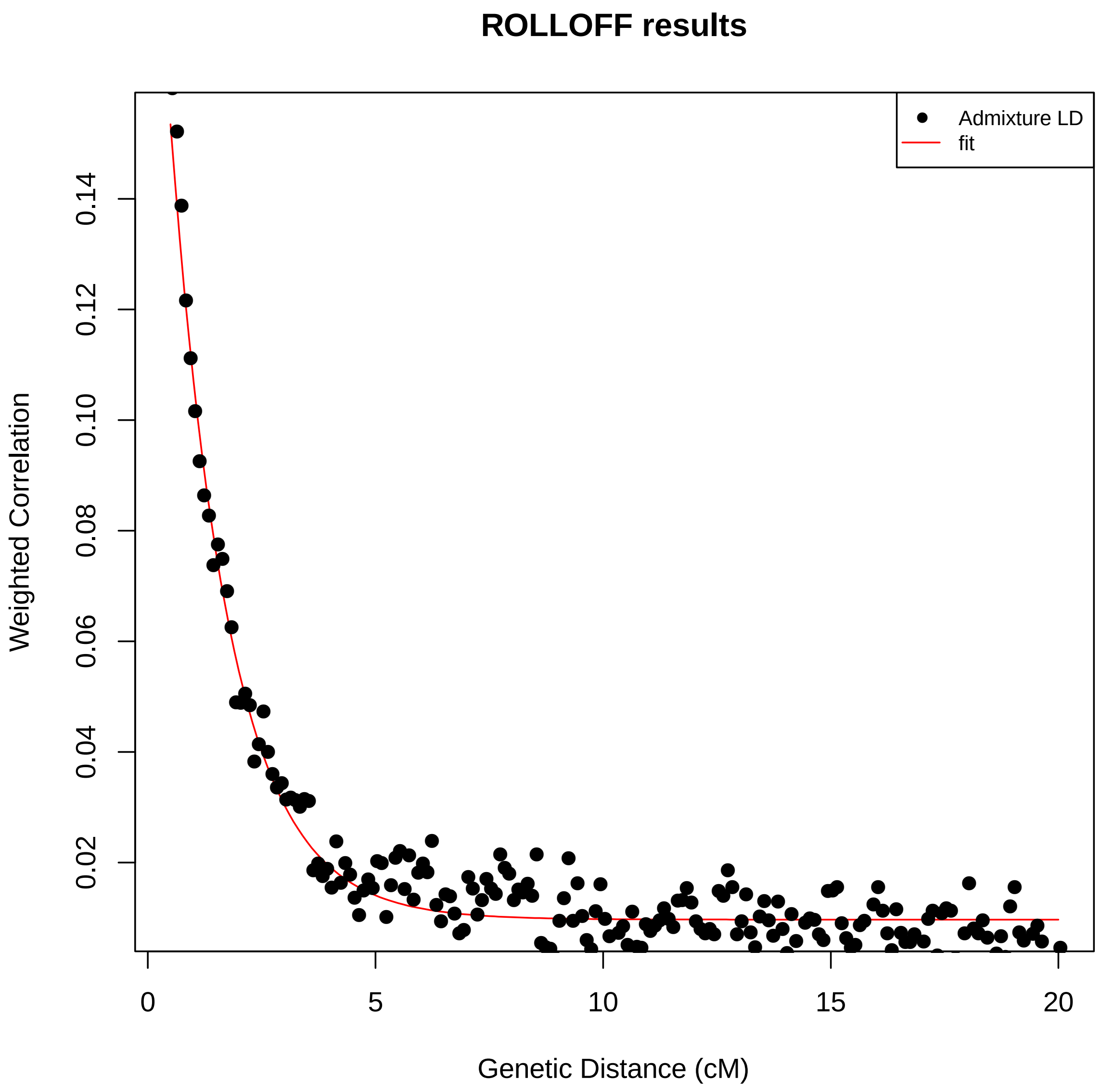

Figure S2. Decline of linkage disequilibrium.
The correlation between a statistic of linkage disequilibrium of a pair of markers and a weight which reflects their allele frequency differentiation in the ancestral populations was plotted as a function of genetic distance (cM). Admixture date was estimated by fitting an exponential distribution to this decline curve in ROLLOFF.

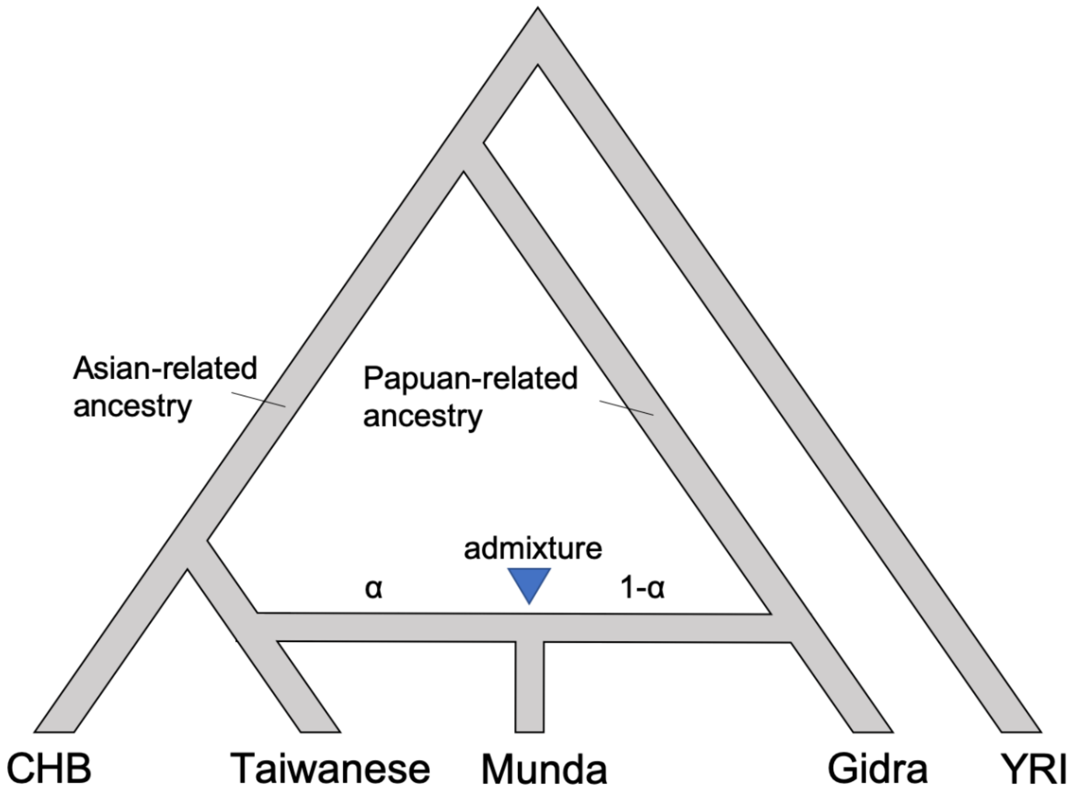

Figure S3. A phylogeny used for f4-ratio estimation.
CHB and Taiwanese are a clade and Gidra and YRI are increasingly distant outgroups in this phylogeny. The proportion of Asian-related ancestry in Munda was α.

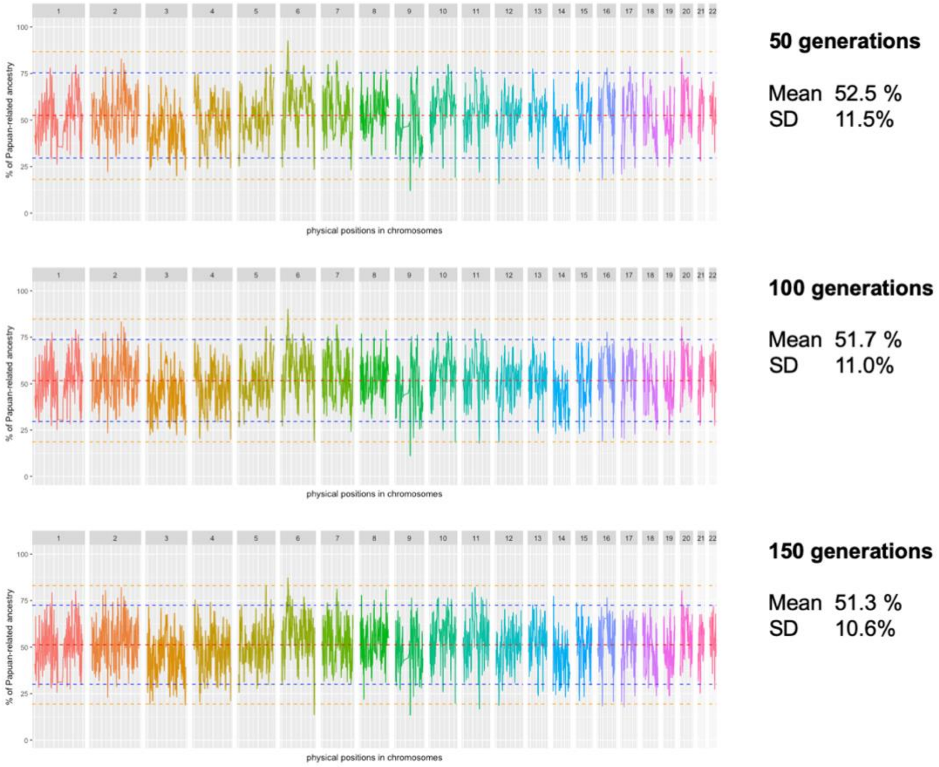

Figure S4. Average Papuan-related ancestry across Munda genomes estimated using ELAI assuming the date of admixture as 50, 100 and 150.
Red dashed line represents the genome-wide mean. Blue and orange dashed lines represent ± 2 SD and ± 3 SD from the mean, respectively. The genome regions above the upper orange line and the lower orange line were regarded as the high Papuan- and Asian- related ancestry regions, respectively.

a

Generations	Mean-3sd(%)	Position	Proportion of Papuan ancestry (%)	MIN_position	Representative Genes
50	18.13	chr9:74635710-76433950	12.01-18.01	chr9:75695677	C9orf57, GDA, 5S_rRNA, AK095210, ZFAND5, TMC1, **ALDH1A1, ANXA1**
		chr12:14948762-16273615	15.76-18.12	chr12:15370878	WBP11, C12orf60, SMCO3, ART4, MGP, C12orf60, ERP27, ARHGDIB, PDE6H, **RERG**, PTPRO, EPS8, STRAP, DERA
		chr16:23050015-23324026	17.86-17.96	chr16:23050015, chr16:23050120, chr16:23050867	**USP31**, SCNN1G, SCNN1B
77	17.39	chr6:168506547-169034278	15.31-17.26	chr6:168978201, chr6:168980936	DACT2, **SMOC2**
		chr9:74835176-76273853	13.96-17.39	chr9:75695677, chr9:75705507	GDA, 5S_rRNA, AK095210, ZFAND5, TMC1, **ALDH1A1, ANXA1**
		chr11:84112590-84386276	16.35-17.36	chr11:84370674, chr11:84371133	**DLG2**
100	18.59	chr9:74659028-76433395	11.06-18.41	chr9:75810393	C9orf57, GDA, 5S_rRNA, AK095210, ZFAND5, TMC1, **ALDH1A1, ANXA1**
		chr10:131086824-131427717	18.33-18.59	chr10:131146611	**MGMT**
		chr11:84222437-84380621	17.9-18.30	chr11:84371133	**DLG2**
		chr12:15349441-15474823	18.43-18.57	chr12:15370878	**RERG**
150	19.43	chr3:13642870-13647142	19.40-19.41	chr3:13647142	**FBLN2**
		chr3:195746771-196315284	18.92-19.31	chr3:195768185	**TFRC**, LINC00885, ZDHHC19, SLC51A, PCYT1A, TCTEX1D2, TM4SF19, AK124973, UBXN7, RNF168, C3orf43, WDR53, FBX045
		chr6:168393286-169110089	13.73-19.28	chr6:168835886, chr6:168835089	HGC6.1.1, KIF25, FRMD1, **DACT2, SMOC2**
		chr9:74682622-76421057	13.46-19.31	chr9:75150031, chr9:75172870	C9orf57, GDA, AK095210, ZFAND5, **TMC1**, ALDH1A1, ANXA1
		chr11:84175049-85657901	16.69-19.29	chr11:84765871	**DLG2,** TMEM126B, TMEM126A, CREBZF, CCDC89, SYTL2, CCDC83
		chr12:15314332-15564674	18.66-19.40	chr12:15370878	**RERG**, PTPRO
		chr16:23036048-23324026	18.27-19.01	chr16:23050015, chr16:23050120, chr16:23050867	**USP31,** SCNN1G, SCNN1B
		chr17:15206970-15308844	17.81-18.94	chr17:15248095, chr17:15248353, chr17:15248399	**TEKT3**

b

Generations	Mean+3sd(%)	Position	Proportion of Papuan ancestry (%)	MAX_position	Representative Genes
50	86.83	chr6:31058340-33954567	86.84-93.03	chr6:32430975	HLA class II genes
77	85.35	chr6: 32233886-33976742	85.47-88.11	chr6:32430975	HLA class II genes
100	84.77	chr6:31920687-33807091	84.82-90.17	chr6:32430975	HLA class II genes
150	83.09	chr6:32341367-33831672	83.10-87.325	chr6:32430975	HLA class II genes

Figure S5. Genomic regions showing the proportion of Papuan-related ancestry 3 SD away from the mean.
Genomic regions where the proportion of Papuan-related ancestry was lower than mean - 3 SD (a) and higher than mean + 3 SD (b) when setting the generations since the admixture as 50, 77, 100 or 150. The column "MAX_position" or "MIN_position" indicates the positions with the highest or lowest Papuan-related ancestry within each region. The symbols of the genes located in each region were listed in the column "Genes". Gene names written in bold in (a) were the genes adjacent to MIN position. The gray rows in (a) contain the position of the lowest Papuan-related ancestry across genome.

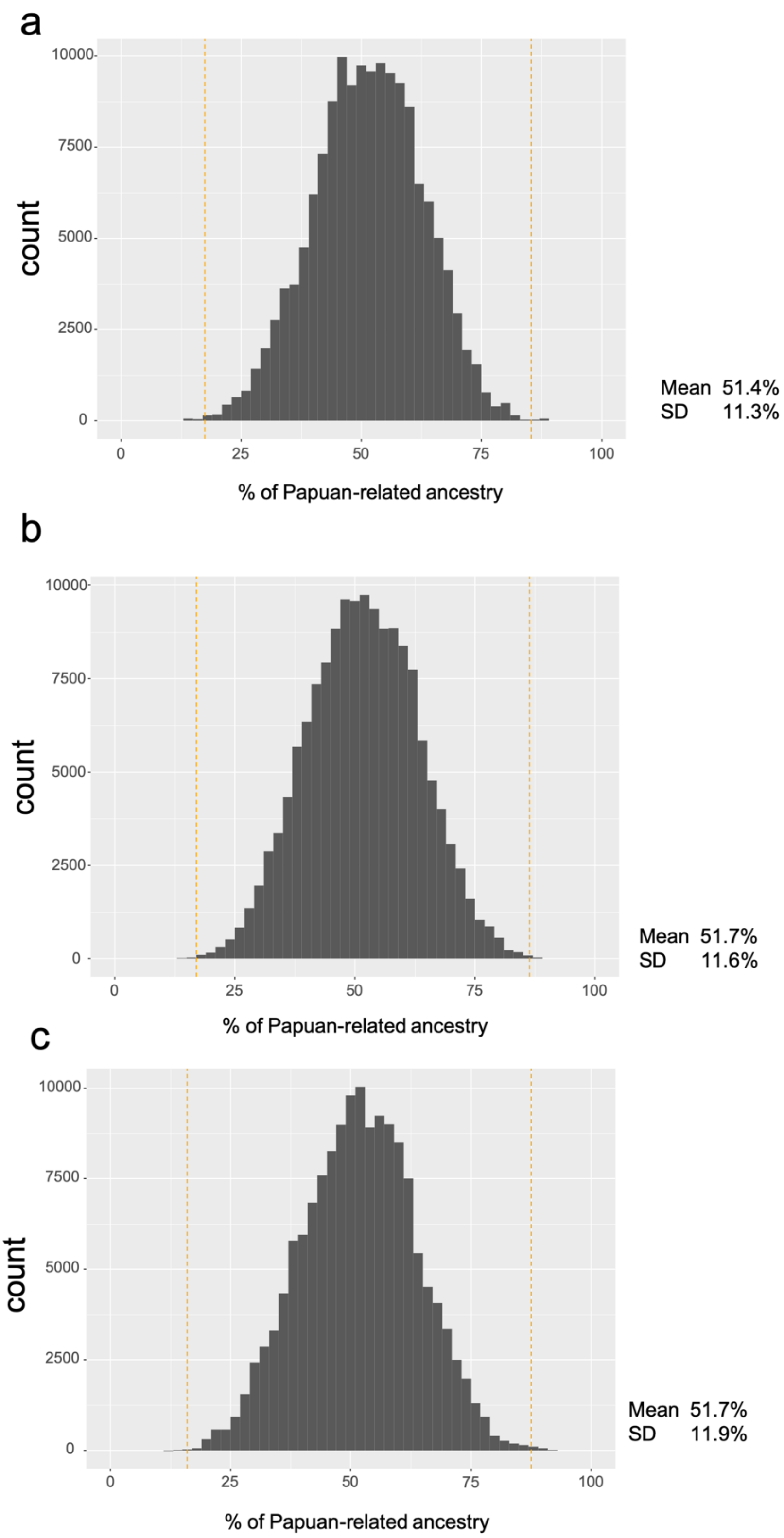

Figure S6. Histogram of the mean (%) of Papuan-related ancestry for each position of every chromosome estimated using genotype data of Munda (a) and simulated data assuming a global recombination rate (i.e. 1.3×10^{-8}/base/ generations) and (b) a recombination rate of the *HLA* region (i.e. 8.5×10^{-9}/base/ generations) (c).

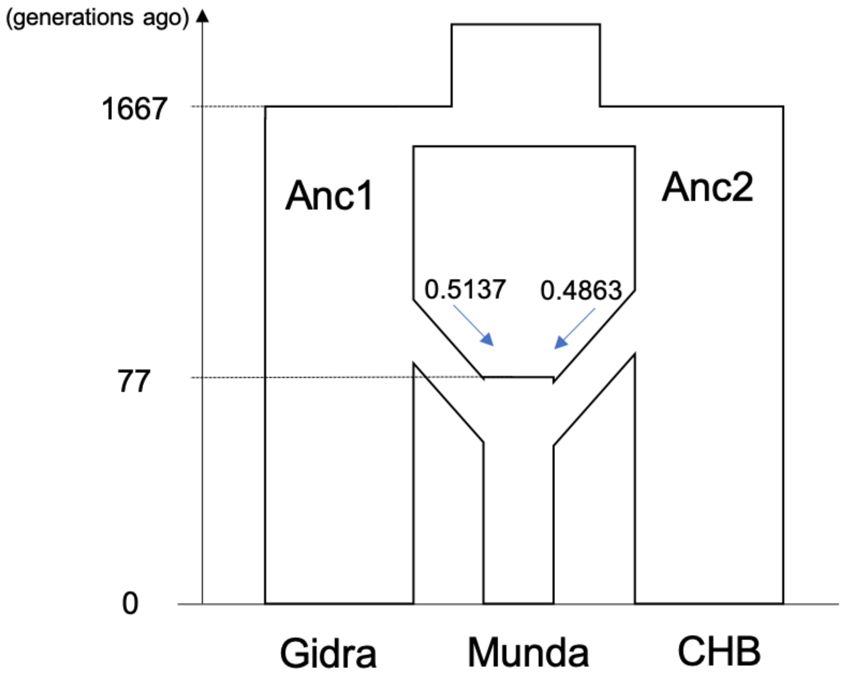

Figure S7. Demographic model reconstructed in coalescent-based simulations.
This model assumed that ancestral populations of Munda (CHB and Gidra as Asian- and Papuan-related ancestors) diverged 1667 generations ago (approximately 50,000 years ago) and admixed 77 generations ago (approximately 2,300 years ago). The admixture rates were 0.5137 for Papuan-related ancestry and 0.4863 for Asian-related ancestry. The size of all populations was set as 1,000.

Supplementary Table

Supplementary Table 1 populations used in this study

Population	Region	N	Ref
Ami	Taiwan	19	Lazaridis et al. 2014; Qin and Stoneking 2015; Pugach et al. 2018
Atayal	Taiwan	16	Lazaridis et al. 2014; Qin and Stoneking 2015; Pugach et al. 2018
Tonga	Polynesia	24	Kimura et al. 2008
Tonga_MS*	Polynesia	7	Qin and Stoneking 2015; Pugach et al. 2018
Ontong_Java	Polynesian outliers	7	Qin and Stoneking 2015; Pugach et al. 2018
RenBel[+]	Polynesian outliers	7	Qin and Stoneking 2015; Pugach et al. 2018
Tikopia	Polynesian outliers	6	Qin and Stoneking 2015; Pugach et al. 2018
Choiseul	Western Solomon Islands	7	Qin and Stoneking 2015; Pugach et al. 2018
Isabel	Western Solomon Islands	8	Qin and Stoneking 2015; Pugach et al. 2018
Kolombangara	Western Solomon Islands	5	Qin and Stoneking 2015; Pugach et al. 2018
Munda	Western Solomon Islands	21	This study
Ranongga	Western Solomon Islands	6	Qin and Stoneking 2015; Pugach et al. 2018
Vella_Lavella	Western Solomon Islands	6	Qin and Stoneking 2015; Pugach et al. 2018
Gela	Eastern Solomon Islands	6	Qin and Stoneking 2015; Pugach et al. 2018
Makira	Eastern Solomon Islands	7	Qin and Stoneking 2015; Pugach et al. 2018
Malaita	Eastern Solomon Islands	6	Qin and Stoneking 2015; Pugach et al. 2018
Russell	Eastern Solomon Islands	3	Qin and Stoneking 2015; Pugach et al. 2018
Savo	Eastern Solomon Islands	7	Qin and Stoneking 2015; Pugach et al. 2018
Santa_Cruz	Eastern Solomon Islands	7	Qin and Stoneking 2015; Pugach et al. 2018
Bougainville_HGDP*	Bougainville Islands	10	Skoglund et al. 2016; Pugach et al. 2018
Bougainville_South	Bougainville Islands	2	Skoglund et al. 2016; Pugach et al. 2018
Buka	Bougainville Islands	8	Skoglund et al. 2016; Pugach et al. 2018
Saposa	Bougainville Islands	9	Skoglund et al. 2016; Pugach et al. 2018
Teop	Bougainville Islands	10	Skoglund et al. 2016; Pugach et al. 2018
Mussau	Mussau Island	10	Skoglund et al. 2016; Pugach et al. 2018
Manus	Manus island	2	Skoglund et al. 2016; Pugach et al. 2018
Kuot_Kabil	New Ireland	9	Skoglund et al. 2016; Pugach et al. 2018
Kuot_Lamalaua	New Ireland	4	Skoglund et al. 2016; Pugach et al. 2018
Madak	New Ireland	9	Skoglund et al. 2016; Pugach et al. 2018
Nailik	New Ireland	9	Skoglund et al. 2016; Pugach et al. 2018

Notsi	New Ireland	9	Skoglund et al. 2016; Pugach et al. 2018
Tigak	New Ireland	10	Skoglund et al. 2016; Pugach et al. 2018
Ata	New Britain	8	Skoglund et al. 2016; Pugach et al. 2018
Baining_Malasait	New Britain	5	Skoglund et al. 2016; Pugach et al. 2018
Baining_Marabu	New Britain	10	Skoglund et al. 2016; Pugach et al. 2018
Kol_New_Britain	New Britain	2	Skoglund et al. 2016; Pugach et al. 2018
Kove	New Britain	18	Skoglund et al. 2016; Pugach et al. 2018
Lavongai	New Britain	15	Skoglund et al. 2016; Pugach et al. 2018
Mamusi	New Britain	20	Skoglund et al. 2016; Pugach et al. 2018
Mamusi_Paleabu	New Britain	6	Skoglund et al. 2016; Pugach et al. 2018
Mangseng	New Britain	6	Skoglund et al. 2016; Pugach et al. 2018
Melamela	New Britain	10	Skoglund et al. 2016; Pugach et al. 2018
Mengen	New Britain	10	Skoglund et al. 2016; Pugach et al. 2018
Nakanai_Bileki	New Britain	10	Skoglund et al. 2016; Pugach et al. 2018
Nakanai_Loso	New Britain	7	Skoglund et al. 2016; Pugach et al. 2018
Sulka	New Britain	18	Skoglund et al. 2016; Pugach et al. 2018
Tolai	New Britain	20	Skoglund et al. 2016; Pugach et al. 2018
Gidra	New Guinea	24	Kimura et al. 2008
New_Guinea*	New Guinea	19	Qin and Stoneking 2015; Pugach et al. 2018
New_Guinea_HGDP*	New Guinea	16	Lazaridis et al. 2014; Pugach et al. 2018
Papuan_Central_Province	New Guinea	5	Qin and Stoneking 2015; Pugach et al. 2018
Papuan_Eastern_Highlands	New Guinea	4	Qin and Stoneking 2015; Pugach et al. 2018
Papuan_Gulf_Province	New Guinea	3	Qin and Stoneking 2015; Pugach et al. 2018

*Buogainville HGDP, New_Guinea_HGDP and Tonga_MS are the same populations with Nasioi, Papuan and Tongan in Pugach et al. (2018), respectively. New_Guinea is comprised of Papuan_Eastern_Highlands, Papuan_Huli and Papuan_Mendi_Kewa in Pugach et al. (2018).

[+]Renbel is a combined sample from the islands of Renell and Bellona nature
